# The urgency of supportive care for sleep after cancer

**DOI:** 10.1093/sleep/zsad120

**Published:** 2023-04-19

**Authors:** Joshua F Wiley

**Affiliations:** Turner Institute for Brain and Mental Health and School of Psychological Sciences, Monash University, Melbourne, Victoria, Australia; Peter MacCallum Cancer Centre, Melbourne, Victoria, Australia

In the Unites States alone, nearly 2 million new cancer cases are estimated to occur in 2023 [1], and it was estimated that there were over 18 million people with a history of cancer alive in 2022 [2]. Although global estimates of the number of people alive with a history of cancer are challenging, incidence data from the Global Burden of Disease Study 2019 show an estimated 23.6 million new cancer cases worldwide in 2019 [3]. These statistics highlight the magnitude of challenge faced in managing and supporting sleep in people after a cancer diagnosis.

Given the number of people with cancer, there is a great need to understand how to efficiently allocate resources to optimally support sleep. Considering that common cancer-related treatments such as surgery and steroids, commonly used during chemotherapy, negatively impact sleep, it is plausible that sleep may improve, even without intervention, following completion of cancer treatment. Indeed, research has shown that related symptoms such as fatigue demonstrate marked improvement in the first 2 years after treatment completion [4]. However, little research has examined the longitudinal trajectories of sleep after completion of primary cancer treatment.

The study in this issue by Chan et al. [5] addresses this critical gap answering whether sleep problems naturally resolve or persist after cancer treatment. They recruited a large sample of adults aged 18–85 years who had completed primary cancer treatment within 6 months, and these were assessed 6 times over 2 years. People commonly experience different symptom trajectories. Such heterogeneity often is lost in reporting an average trajectory. Chan et al. [5] overcome this limitation by using sophisticated statistical models, latent growth mixture models, that are able to identify distinct classes of trajectories thus quantifying heterogeneous patterns of change over time. They found the most consistent evidence for two trajectories of sleep after cancer treatment completion: “stable good sleep,” comprising 69.7% of participants, and “persistently high sleep disturbance,” comprising 30.3% of the participants. Interestingly, both trajectories were stable and did not evidence any clinically meaningful improvement or worsening over the 2-year study.

These results highlight that for a substantial number of people (30.3%) sleep problems do not resolve after completion of cancer treatment. That substantial poor sleep was stable and persistent over a 2-year period underscores that only treating the cancer and hoping that troubling sleep problems will simply resolve on their own after treatment completion is inadequate. Although nearly 70% of people fell into the class labeled as “stable good sleep,” it is notable that in this group, the average Pittsburgh Sleep Quality Index [6] hovered just below or just above a score of five, a cutoff indicative of poor sleep. Thus, even amongst the majority of stable good sleepers, many may be expected to have at least some level of sleep disturbance, albeit substantially lower than the 30.3% of people with persistently high sleep disturbance. Chan et al. [5] other key finding were that psychological factors (i.e. levels of depressive symptoms, intrusive thoughts about cancer, hyperarousal towards cancer, and avoidant thoughts about cancer) but not physical symptom distress predicted which sleep trajectory class people were likely to experience, findings consistent with other studies during cancer treatment [7].

Chan et al. [5] provide critical evidence that sleep problems persist for a substantial number of people after cancer treatment. The inclusion of a variety of cancer types and a large sample size both increase the likelihood that these results are reliable and may generalize across cancer types. The findings highlight the urgent need for routine, supportive care interventions to be offered and delivered to people with cancer. Although this is a daunting task, given the millions of people with cancer, there are numerous efforts underway to develop efficient and scalable approaches to managing sleep after cancer. For example, a Pan-Canadian practice guideline proposed three care pathways based on brief assessments ranging from psychoeducation to specialist intervention [8]. Chan et al. [5] findings that even those people in the “stable good sleep” trajectory hovered around the Pittsburgh Sleep Quality Index threshold for poor sleep supports the Pan-Canadian guideline recommendation that all people with cancer receive preventive and supportive education for sleep [8]. There is also growing evidence that stepped-care models to support sleep after cancer are feasible [9] and emerging evidence that stepped-care results in non-inferior outcomes compared to standard full-intensity sleep interventions [10]. Chan et al. [5] results also highlight how sleep does not exist in isolation and that poor sleep may be exacerbated by other factors, particularly psychological symptoms. High-quality, holistic care [11] including supportive care for sleep and other symptoms is necessary if we are to meet the needs of people with cancer. Chan et al. [5] have provided high-quality data on the need, now it is up to the sleep field to work with people who have experienced cancer, the community, and health services to respond for a better sleep and a brighter tomorrow.
